# An unusual case of aggressive systemic mastocytosis mimicking hepatic cirrhosis

**DOI:** 10.7497/j.issn.2095-3941.2014.02.009

**Published:** 2014-06

**Authors:** Xiao-Yang Zhang, Wei-Hua Zhang

**Affiliations:** ^1^Department of Pathology, Second Hospital of Tianjin Medical University, Tianjin 300211, China; ^2^Department of Dermatology, Traditional Chinese Medical Hospital of Rizhao, Rizhao 276000, China

**Keywords:** Aggressive systemic mastocytosis (ASM), hepatomegaly, hepatic fibrosis, hepatic cirrhosis

## Abstract

Hepatic involvement in aggressive systemic mastocytosis (ASM) is relatively common, and the main clinical features of this disease include hepatomegaly, portal hypertension, ascites, and fibrosis. Cirrhosis is a rare ASM symptom. We report an ASM case that initially mimicked cirrhosis based on clinical and radiographic analyses. The portal tract was expanded by mononuclear inﬂammatory cells, and an increase in collagen amount was observed in routine histological sections of the biopsied liver. A diagnosis of systemic mastocytosis (SM) was made after ancillary tests for mast cells using bone marrow aspirates. Extensive involvement of the liver and gastrointestinal tract was observed. Clinicians and pathologists need to consider ASM as a diagnosis or differential diagnosis in a clinical case of cirrhosis with unknown etiology. The diagnosis can be confirmed or disregarded by immunohistochemical staining and molecular analysis.

## Introduction

Systemic mastocytosis (SM) is characterized by the presence of a heterogeneous group of disorders. These disorders involve the growth and accumulation of morphologically and immunophenotypically abnormal mast cells in at least one extracutaneous organ with or without skin lesions. Aggressive systemic mastocytosis (ASM) is a subtype of SM. An SM diagnosis needs to fulfill the WHO^1^ diagnostic criteria, which include one major and one minor criterion or at least three minor criteria. The major criterion is the presence of multifocal dense infiltrates of mast cells in aggregates of 15 cells or more. Such aggregation can be confirmed using immunohistochemical stains for either c-kit or tryptase in sections of bone marrow and/or other extracutaneous tissue(s). Minor criteria include the following: (1) more than 25% of the total number of mast cells are immature or show atypical morphology; (2) mast cells aberrantly express CD2 and/or CD25 (normal mast cells are typically negative for these lymphocyte markers); (3) serum/plasma tryptase level persistently exceeds 20 ng/mL; and (4) the presence of a codon 816 of KIT mutation in the peripheral blood, bone marrow, or lesional tissue. Moreover, the diagnosis of ASM can be made when one or more “C” findings are present. “C” findings include the following: (1) anemia (Hb<10 g/dL), thrombocytopenia (<100,000/mm^3^), and neutropenia; (2) hepatopathy with ascites or portal hypertension; (3) splenomegaly with hypersplenism; (4) malabsorption with weight loss; and (5) osteolysis with pathological bone fractures.

ASM exhibits highly variable clinical characteristics, and skin lesions are often absent. Overlapping symptoms and heterogeneous clinical scenarios make early and accurate diagnosis extremely difficult. Patients are frequently undiagnosed or misdiagnosed. The primary symptom of ASM is the pathological accumulation of mast cells in various tissues. The liver is frequently involved, but only a minor percentage of ASM patients develop portal hypertension and ascites. Cirrhosis symptoms are rarer. The liver is rarely biopsied and examined unless significant organ dysfunction has developed. In this paper, we report a rare case of ASM without skin lesions. Only hepatic and gastrointestinal tract symptoms were observed in the patient. This report prompts pathologists and gastroenterologists to consider ASM as a differential diagnosis in patients with hepatic and gastrointestinal tract diseases of unknown etiology. The study was approved by the institutional ethics committee.

## Case report

A 62-year-old female patient was referred to the digestive system department of our hospital because of progressive abdominal distension for 1.5 years and edema of bilateral lower limbs for one month. The patient presented with the following symptoms upon admission: malaise, weight loss (10 kg), intermittent upper abdominal pain, watery diarrhea (three to four times daily), oliguria (500 mL/day), and transient skin flushes. The patient’s blood pressure declined from 120/90 mmHg to 100/70 mmHg. She did not receive any therapy before admission. Her medical history revealed that she had tuberculosis for 40 years and other unremarkable symptoms.

After physical examination, abdominal ultrasonography, and computed tomography (CT) scan, the following symptoms were detected: liver palm (palmar erythema), pot belly, bilateral lower limb edema, hepatomegaly (4 cm below the costal margin), mild splenomegaly, ascites, and nodular infiltration of the liver. Skin lesions, rashes, and superficial lymphadenopathy were absent.

Results of the laboratory examination performed at admission revealed a white blood cell count of 5.4×10^9^/L with a normal differential count, hemoglobin level of 107 g/L, and platelet count of 249×10^9^/L. The patient had a serum sodium level of 126 mmol/L (normal: 137-150 mmol/L), albumin level of 31.6 g/L (normal: 37-53 g/L), aspartate aminotransferase level of 12.9 U/L (normal: 0-40 U/L), alanine aminotransferase level of 13.2 U/L (normal: 0-40 U/L), gamma-glutamyltransferase level of 93 U/L (normal: 0-50 U/L), alkaline phosphatase level of 148 U/L (normal: 42-128 U/L), total bilirubin level of 6.5 µmol/L (normal: 0-20 µmol/L), direct bilirubin level of 1.4 µmol/L (normal: 1.7-6.8 µmol/L), and prothrombin time of 24.3 s (normal: 12-16 s). The tumor marker CA125 was present at a level of 207.6 U/mL (normal: <35 U/mL). Serological testing for hepatitis A, B, and C and HIV showed negative results. Autoimmune markers, other tumor markers, and blood and fecal cultures were negative.

A preliminary diagnosis based on clinical symptoms and laboratory examination results of the patient was made by the physician as follows: “hepatic cirrhosis, decompensated phase, however, etiology was not clear. In addition, given the tumor marker CA125 was in high level, exclude other abdominal or gynecological tumor was necessary.”

The patient underwent upper and lower gastrointestinal endoscopy three times during hospitalization because of persistent and intermittent diarrhea. She also underwent a liver biopsy. Subsequently, the patient developed anemia (hemoglobin level of 98 g/L). Bone marrow aspiration was performed, and results show an abundance of atypical hypogranulated round or spindle-shaped cells. The abundance of such cells conﬁrmed the presence of mast cell differentiation. Further immunohistochemical staining was performed on cells. Diffuse CD25 and CD117 in tryptase-positive mast cells infiltrated the gastric and colonic mucosa. Sheets of mast cells were present in the portal tract of the liver.

**Figure 1:**
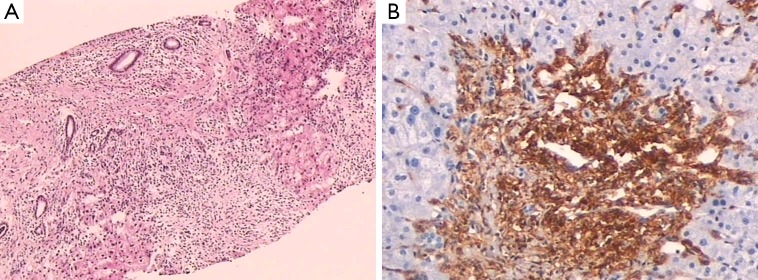
Microscopic images of the liver. (A) Liver biopsy highlighted an extinction area comprising a large amount of spindled mast cells, fibrous tissue, entrapped hepatocytes, ductular reactions, and a portal spanning the width of the needle’s core (H&E ×40); (B) mast cells were strongly positive for CD117 (IHC ×100).

The liver biopsy highlighted an area of extinction comprising a large amount of spindled mast cells, fibrous tissue, entrapped hepatocytes, ductular reactions, and a portal spanning the width of the needle’s core (**Figure 1A**). Mast cells were positive for CD117 (**Figure 1B**) and CD25, but negative for CD30. The mast cells were loosely scattered in the hepatic sinusoids within the hepatic parenchyma.

**Figure 2:**
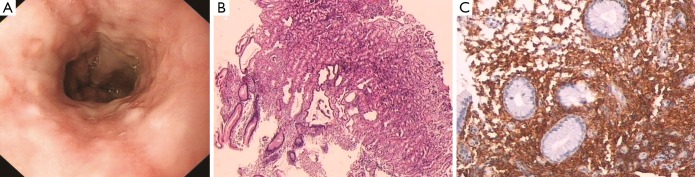
(A) Endoscopic image of the stomach showing erythema, superficial erosions, and nodularity without ulceration; (B) microscopic appearance of the gastric mucosa showing erosion, dilated and distorted gland architecture, increase in mast cells with chronic inflammation, and cell infiltration in the lamina propria (H&E ×20); (C) mast cells were highlighted by positive CD117 (IHC ×100).

A diffuse hyperemia with superficial erosions was present. The mucosa showed a slightly raised appearance, which mimics the proliferative polyp found by upper and lower gastrointestinal endoscopy (**Figure 2A**). The results of histological analysis were as follows. Mucosal erosion and gland expansion and distortion were observed. Diffuse spindled mast cell infiltration in the gastric and colonic lamina propria was characterized by cells with small, elongated, and baculiform nuclei, inconspicuous nucleoli, and pale cytoplasm (**Figure 2B**). These cells were admixed with some lymphocytes and plasma cells. The tumor cells were positive for CD117 (**Figure 2C**), and showed abnormal surface molecular expression of CD25. However, tumor cells were negative for CD30.

These ﬁndings were fully consistent with ASM according to the WHO criteria, and a diagnosis of ASM with extensive involvement of the liver and gastrointestinal tract was made. The patient received H_1_ and H_2_ histamine antagonists with improvement in diarrhea. The patient was discharged upon the demand of her family and the patient herself. The patient died after one month.

## Discussion

Hepatic involvement in SM is common. Evidence of hepatic involvement includes hepatomegaly, portal hypertension, ascites, fibrosis, cirrhosis, liver failure secondary to SM, and abnormal liver test results. The main characteristics of SM patients with hepatic involvement according to the literature are summarized in **Table 1**^2^^-^^12^. SM patients frequently showed the following gastrointestinal and hepatic symptoms: hepatomegaly (90%), splenomegaly (79%), diarrhea (54%), abdominal pain (50%), and ascites (37%).

**Table 1:** Main characteristics of patients suffering from SM with hepatic involvement. Data were collected from the current study and previous studies

Characteristics	Yam^2^ (1986) *n*=13 (%)	Horny^3^ (1989) *n*=131 (%)	Mican^4^ (1995) *n*=25 (%)	Kyriakou^5^ (1998) *n*=4 (%)	Cases* (1989 to 2012) *n*=9 (%)	Total *n*=182 (%)
Age, yrs	-	-	-	47-75	35-73	35-75
Male:Female ratio	-	-	-	2:2	4:5	6:7
Abdominal pain	6 (46)	-	-	-	5 (55)	11/22 (50)
Diarrhea	7 (54)	-	-	-	5 (55)	12/22 (54)
Ascites	5 (38)	-	5 (20)	2 (50)	7 (78)	19/51 (37)
Hepatomegaly	10 (77)	131 (100)	10 (40)	4 (100)	9 (100)	164/182 (90)
Splenomegaly	-	-	17 (68)	4 (100)	9 (100)	30/38 (79)
Lymphadenopathy	-	-	-	0 (0)	3 (33)	3/13 (23)
KIT mutation	-	-	-	-	2/3 (67)	2/3 (67)
Skin lesions	-	87 (66)	-	0 (0)	2 (22)	89/144 (62)
Portal fibrosis	13 (100)	25/77 (32)	19 (76)	-	5/6	62/121 (51)
Cirrhosis	2 (15)	7 (9)	0	0	0/6	9/124 (7)
Eosinophils	-	-	15 (60)	-	2/3	17/28 (61)

*, cases^6^^-^^12^ and present case; -, not mentioned.

Liver biopsy is rarely performed unless significant organ dysfunction has developed. Thus, information on liver histopathological features of SM patients is limited. The main sign of hepatic involvement in SM is the pathological accumulation of mast cells in the liver. Mast cells aggregate predominantly in portal areas. The most common symptoms are portal fibrosis and eosinophilic infiltration. Portal fibrosis was observed in 51% of the total number of cases. Mican *et al*.^4^ observed a direct correlation between mast cell infiltration and fibrosis. Eosinophilic infiltration was observed in 61% of the total number of cases (**Table 1**), and may be correlated with hepatic mast cell burden^13^. The occurrence of portal cirrhosis was relatively rare (7%) (**Table 1**). Kyriakou *et al*.^9^ described three patients with liver biopsy characteristics that were consistent with autoimmune cholangitis. They concluded that SM rarely causes non-cirrhotic portal hypertension, often simulating autoimmune cholangitis and leading to an erroneous diagnosis of liver cirrhosis. Other reported pathological findings included the following symptoms: a dense aggregation of mast cells inﬁltrating the central veins, thereby increasing the thickness of the walls and obliterating luminal structures; muscular hypertrophy around the occluded vessel; subendothelial collagen deposition; and sinusoidal dilatation, centrilobular necrosis, cholestasis, and cholangitis^2^^-^^9^^,^^13^^,^^14^.

Despite significant hepatic involvement, normal values are obtained by the usual liver tests (except for the alkaline phosphatase test) in SM patients^13^. Furthermore, hepatic lesion severity is not correlated with the size of the liver or the results of liver function tests^13^. Mican *et al*.^4^ demonstrated that five patients who showed symptoms of ascites or portal hypertension died of complications secondary to SM. However, mild impairment of liver function was observed in these patients. Thus, the development of ascites or portal hypertension is an adverse prognostic marker. The lack of skin involvement also results in negative prognosis for an SM patient.

In the present case, the patient was initially diagnosed with cirrhosis based on the CT and clinical results. Microscopic examination of liver biopsy showed disturbed architecture with portal fibrosis and spindled mononuclear cell infiltration. Common eosinophils were absent. The histological examination results were inconclusive. The abnormal mast cells were difficult to identify, particularly in the presence of other cells, such as lymphocytes, macrophages, and eosinophils. The mixture of cells simulated changes in chronic active hepatitis. Thus, a special stain was necessary to distinguish the cells. Normal mast cells typically expressed two characteristic surface markers, namely, the CD117 antigen and IgE. However, these markers were not speciﬁc. CD25 and/or CD2 are invariably expressed in neoplastic mast cells from SM patients^10^. More recent data suggested that most neoplastic mast cells in indolent SM cases are usually negative for CD30. By contrast, CD30 is expressed abundantly in the cytoplasm of most mast cells in patients with advanced SM, such as ASM and mast cell leukemia^11^. Some authors considered that CD30 expression may help in conﬁrming the diagnosis of advanced SM and excluding other potential neoplasms^15^. Unexpectedly, CD30 was not expressed in the current case.

## Conclusion

SM diagnosis can be challenging especially in the absence of typical skin manifestations. Diagnosis is difficult even if a patient shows symptoms of aggressive disease, impaired liver function, hepatomegaly, ascites, and cirrhosis. A diagnosis of SM must be considered during routine clinical practice. This diagnosis can be confirmed or disregarded using immunohistochemical markers, such as tryptase, CD117, and CD25, in combination with molecular analysis (KITD816V) and laboratory parameters (i.e., serum tryptase).
